# Sex-specific differential gene expression during stolonization in the branching syllid *Ramisyllis kingghidorahi* (Annelida, Syllidae)

**DOI:** 10.1186/s12864-025-11587-w

**Published:** 2025-04-25

**Authors:** Guillermo Ponz-Segrelles, Thilo Schulze, Kohei Oguchi, Daisuke S. Sato, Mayuko Nakamura, Yoshinobu Hayashi, Christopher J. Glasby, Toru Miura, M. Teresa Aguado

**Affiliations:** 1 IES El Burgo-Ignacio-Echeverría, 28231 Las Rozas de Madrid, Madrid, Spain; 2 Animal Evolution & Biodiversity, Georg-August-Universität Göttingen, 37073 Göttingen, Germany; 3 Misaki Marine Biological Station, School of Science, The University of Tokyo, Misaki, Miura, Kanagawa 238 - 0225 Japan; 4 Department of Biology, Keio University, Yokohama, Kanagawa 223 - 8521 Japan; 5 Museum and Art Gallery of the Northern Territory, PO Box 4646, Darwin, NT 0801 Australia

**Keywords:** Gemmiparity, Sexual maturation, Reproduction, Schizogamy, Comparative transcriptomics, Annelid physiology

## Abstract

**Background:**

*Ramisyllis kingghidorahi* (Annelida, Syllidae) is one of few annelid species with a ramified body, one anterior end and hundreds of posterior ends. *R. kingghidorahi* belongs to the family Syllidae, whose members reproduce by forming stolons, small autonomous reproductive units, at the posterior end. Molecular mechanisms controlling sexual reproduction are still poorly understood, but previous studies support an important role of the anterior end and stolons. The roles of different body regions during sexual reproduction in a complex branched body where there is only one head but multiple posterior ends, which develop hundreds of simultaneous stolons, have never been investigated. Consequently, we aimed to research the transcriptomic basis of sexual maturation and stolonization in *R. kingghidorahi* by performing differential gene expression analyses.

**Results:**

Transcriptomes were assembled from different body regions (anterior end, midbody, and stolons) of male, female, and non-reproductive individuals. Comparative analyses revealed that body region had a greater impact on gene expression profiles than sex, with the anterior end and stolons showing extensive gene upregulation. Across-sex comparisons revealed sex-specific processes in all body regions, with stolons exhibiting the most differences in differential expression, likely related to gametogenesis and external sexual dimorphism. Fewer genes than expected were differentially expressed in the anterior region, a result for which different possible explanations are discussed. Surprisingly, key genes typically associated with segmentation and metamorphosis, such as Wnt and Hox, showed little differential expression, aligning with recent findings that stolon segments lack a specific segment identity.

**Conclusions:**

This study presents the first transcriptomic data for a branched annelid species and offers new insights into the complex genetic regulation of reproduction in *R. kingghidorahi*. Additionally, it provides the first glimpse into the mechanisms of sexual maturation in branched syllids, which must coordinate stolonization across multiple posterior ends. These findings enhance our understanding of annelid reproductive biology and highlight the need for further research to uncover the physiological and molecular pathways regulating sexual maturation and stolonization in syllids and other annelids.

**Supplementary Information:**

The online version contains supplementary material available at 10.1186/s12864-025-11587-w.

## Background

Annelids of the family Syllidae Grube, 1850 [1] are characterized by two main features: the presence of a proventricle, and the post-embryonic metamorphic changes associated with sexual maturation [2–4]. The proventricle is an apomorphic structure of the digestive tract that lies immediately posterior to the more strongly-cuticularized, partially-eversible axial pharynx, and is composed of a prominent layer of radially-arranged muscle cells [2–6]. During sexual maturation, many syllid species develop reproductive units (stolons) formed by a few segments at the posteriormost tip of the body. These units contain the gonads and gametes and they are detachable from the rest of the body and usually develop characteristic anterior anatomical features like eyes and brains in addition to swimming chaetae, which allow them to swim and find a mating partner independently [6–10]. This process is known as schizogamy or stolonization.

For several decades, the leading hypothesis about the physiological mechanisms regulating sexual maturation in syllids was that stolonization is constantly suppressed by a Stolonization-Inhibiting Hormone (SIH) associated with the proventricle or the proventricle region and, when the right light and temperature conditions are met, a Stolonization-Promoting Hormone (SPH) produced and/or released by the prostomium inhibits SIH and triggers sexual maturation and stolonization [11–15]. However, later research has shown that hormonal control of sexual maturation in annelids is usually significantly more complex [16–24] and, based on this increasing knowledge, gene expression studies have been performed in syllids to further understand the role of the anterior region in the onset and control of stolonization, refining the original hypothesis. Álvarez-Campos et al. [25] found differential expression of genes involved in methylfarnesoate synthesis in stolonizing *Syllis magdalena* Wesenberg-Lund, 1962 [26], and have proposed that it acts together with dopamine and serotonin in regulating sexual maturation and influencing the expression of several genes involved in gametogenesis and other reproduction-related processes. Similarly, Ponz-Segrelles et al. [27] discussed the issue of sex determination and its possible relation with irreversible gene expression changes during the worms’ lives. More recently, Nakamura et al. [24] studied the expression patterns of several candidate genes, including stem cell markers and Hox genes, during stolonization in *Megasyllis nipponica* (Imajima, 1966) [28]. Yet, there is still much to learn about the mechanisms involved in the many changes associated with sexual maturation in Syllidae. For example, despite the extreme metamorphic changes that take place, there is still no information concerning the mechanisms involved in stolon formation, which must include, but may not be limited to, axis patterning, nervous system and eye development, chaetae formation, gonad development, gametogenesis, and behaviour.

On top of this, syllid reproduction includes a variety of different types of stolonization (i.e. scissiparity and several kinds of gemmiparity) [29], with those present in the Ribbon Clade (sensu [29, 30]) being the most remarkable [7, 29]. This clade includes species that produce only one stolon per reproductive event (scissiparity), but also others that simultaneously produce multiple stolons (gemmiparity), which may be clustered in bunches attached ventrally at the posteriormost one or two parental segments, or individually attached to different posterior segments [31–34]. However, the most striking species of the Ribbon Clade are those with a branched body [29, 35, 36], something that was initially thought to be restricted to a single species, but is now known in three, presumably related different species from a wide geographic range and will likely be described for related, newly-described species in the future [37].

**Fig. 1:**
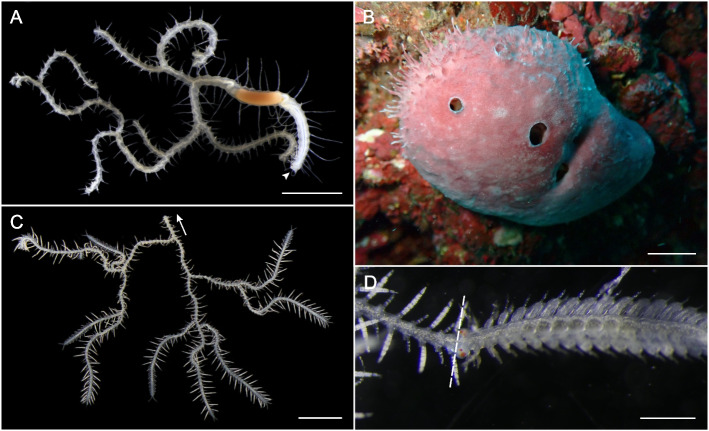
Stereomicroscopy images of living specimens of *Ramisyllis kingghidorahi* and in situ underwater photography of its host sponge *Petrosia* sp. **A**, Fragment of the anterior region including the prostomium, proventricle, and first branches, dorsal view. Arrowhead points to the anterior end. **B**, Host sponge *Petrosia* sp. in its natural habitat [modified from 54]. **C**, Fragment of a *R. kingghidorahi* specimen including several posterior ends. Arrow points towards the direction of the anterior end, which is missing. **D**, Developing male stolon attached to its parental stalk. Dashed line indicates the boundary between the stalk (left) and the stolon (right). Scale bars: 2 mm (**A**, **C**); 1 cm (**B**); 250 µm (**D**)

Branched syllids obligatorily inhabit sponges and exhibit a body plan unique among the annelids (Figs. 1A, B). These animals show a single anterior end with a regular head and foregut that is followed by a ramified body in which the body recursively branches laterally [35–37] (Figs. 1A, C). Lateral branches are known to include all longitudinal internal organs and are, therefore, considered to be complete bifurcations of the anteroposterior axis of the animal [38]. Notably, such branched bodies result in the existence of hundreds or thousands of complete posterior ends, each of them potentially capable of stolonization [36]. This way, a single stolonizing specimen of branched syllid can produce hundreds of stolons simultaneously [36, 37]. Stolons (Fig. 1D) show sexual dimorphism in their external anatomy, and each sexually mature specimen produces either male or female stolons exclusively during each stolonization cycle [35–38]. Unfortunately, branched syllids have proven to be very elusive and have only been occasionally found in very limited places since they were first discovered [35–37]. Thus, due to their specific habitats, difficult accessibility for sampling, the challenges of studying symbiotic organisms both in the lab and field, and the many difficulties faced in the past when trying to obtain sequence data, not much is known about these enigmatic animals. However, the peculiar anatomy of branched syllids of the genus *Ramisyllis* have been recently explored in detail, and it has been proposed that physiological innovations must also be present in these species since basic processes like digestion or blood circulation are likely to be affected by their branched bodies [38].

As explained, syllids show great diversity in relation to the metamorphic changes associated with sexual maturation, including extremes such as those described for *Ramisyllis*. However, up to now, all available gene expression information comes only from species producing stolons through scissiparity [24, 25, 27, 39]. Thus, the true complexity and variability of the many mechanisms that must be involved in controlling sexual maturation in syllids remains virtually unknown. Studying these mechanisms in syllids with different reproductive modes is particularly interesting, as it is unclear whether the anterior end and stolons, known to regulate sexual maturation in scissiparous syllids, also function similarly in branched syllids, which have numerous posterior ends requiring stolonization control. Considering the previous studies and proposed stolonization control hypotheses [7, 24, 25], the single head of these animals must play a highly important role in regulating the reproductive processes occurring in hundreds of posterior ends. Thus, the aim of this study is to characterize gene expression changes associated with stolonization and examine what regions are involved in reproductive processes in such huge and complex branched bodies.

Here we present an exploratory analysis of gene expression in stolon-bearing male and female specimens of the branched syllid *Ramisyllis kingghidorahi* Aguado et al., 2022 [37]. We used transcriptome-based differential gene expression analyses to compare the expression profiles from three body regions (anterior ends, midbody fragments, and stolons) in reproductive males and females as well as non-reproductive individuals, and focused on the expression patterns of those transcripts that we identified as potentially involved in processes related to sexual maturation/reproduction.

## Materials and methods

### Sampling

Specimens of *Ramisyllis kingghidorahi* and their host *Petrosia* sp. sponges (Fig. 1A-D) were collected in Sado Island (Japan) in October 2019 and 2022. Specimens were collected by scuba diving at Shukunegi Point, at the southern tip of the island (37°48′17.1"N, 138°14′25.1"E) [37]. Sponges of the genus *Petrosia* hosting *R. kingghidorahi* (Fig. 1B) were mainly found attached to vertical rock walls 10–15 m deep. All *Petrosia* sp. specimens were collected in one piece and placed in plastic bags filled with natural seawater. After collection, specimens were carried to Sado Marine Biological Station (Niigata University) and placed in plastic trays completely covered with constantly-running natural seawater. *Ramisyllis kingghidorahi* specimens were removed from their host sponges as previously described [36]. Stolon-bearing specimens were selected and identified as male or female based on stolon dimorphism (Fig. 3). Extracted pieces were sorted into three tissue types (anterior regions including the prostomium and proventricle, midbody segments, and stolons; Fig. 4) and immediately preserved in RNAlater (Ambion, Darmstadt, Germany). Three replicates were prepared for male and female stolon samples, four replicates were prepared for the midbody tissue categories of males and females and six replicates were prepared for the non-reproductive individuals. In the case of anterior ends, three replicates were prepared for male and non-reproductive animals, and two were prepared for female specimens. Due to the inherent difficulty of collecting *R. kingghidorahi* specimens, the sample size in this study is smaller than recommended, which may cause an increased rate of false positives [40]. Some replicates are not completely independent as they come from the same individual, including two sets of two samples coming from the same body region of the same individual (SA20 and SA21 for male stolons and SA50 and SA52 for female midbodies; Supplementary File S1). This strategy resulted in a total of 28 samples from 15 unique individuals. Sample information of the 28 sequenced samples can be seen in Supplementary File S1.

### Library construction, sequencing, assembly, and annotation

Total RNA was extracted from the preserved specimens with Isogen reagent standard protocol (Nippon Gene, Tokyo, Japan). Co-extracted genomic DNA was digested with DNase (TURBO DNA-free™ kit, Ambion, Austin, TX, USA) and RNA samples were purified by Magnetic beads (AMPure XP Beckman Coulter, Brea, USA). RNA quality was then assessed by RNA ScreenTape with an Agilent TapeStation 2200 (Santa Clara, CA, USA). Library construction and sequencing were outsourced to Eurofins Genomics Inc. (Tokyo, Japan) and Genome-Lead Inc. (Kagawa, Japan), where all samples were sequenced with an Illumina NovaSeq6000 machine (Illumina, San Diego, CA, USA).

Raw RNA-seq sequences of each sample were quality-checked with FastQC v0.11.5 (http://bioinformatics.babraham.ac.uk/projects/fastqc/), and Trimmomatic v0.38 [41] was used for filtering and trimming low quality reads. Reads from all 28 samples were pooled for reference transcriptome assembly following Trinity’s v2.15.0 pipeline [42, 43]. To reduce redundancy and improve downstream differential expression analyses, the transcripts in the assembly were clustered with mmseqs2 v13.45111 [44]. Clustering was carried out using the linclust workflow (https://github.com/soedinglab/mmseqs2/wiki) with target coverage mode enabled, minimum coverage set to 80%, and minimum sequence identity set to 95% [45]. Transcriptome quality and completeness were assessed before and after clustering with the *TrinityStats.pl* script of Trinity and BUSCO v5.4.3 [46]. Furthermore, supertranscripts of the unfiltered assembly were created using the designated Trinity script; then its completeness was evaluated with BUSCO. Once the transcriptome had been assembled and filtered, open reading frame detection and protein-coding region prediction were carried out with TransDecoder v5.5.0 (https://transdecoder.github.io/). Protein predictions were then used for functional annotation of each transcriptome following Trinotate’s v3.2.2 pipeline (http://trinotate.github.io). The most similar sequence for each transcript was identified with BLASTx v2.12.0 + [47], while homologs for predicted protein sequences were found with BLASTp, both using the UniProtKB/Swiss-Prot database. Furthermore, conserved protein domains were identified by searching the predicted protein sequences against the Pfam database via HMMER v3.1b2 (http://hmmer.org) and signal peptides were predicted using signalP v4.1 [48]. Gene ontology assignments were retrieved for downstream analyses by loading the performed homology searches and predictions to the Trinotate SQLite boilerplate database. Annotation success was evaluated based on Annotation Units as previously described [27].

### Differential expression

Nine differential gene expression comparisons were made: one including all samples and considering their body region and sex condition, three comparing the same body region for different sex conditions (female, male, and non-reproductive), three comparing different body regions (anterior end, midbody, and stolon) with samples of the same sex, one including all samples but considering only the body region (regardless of sex), and one including all samples considering only the sex condition (regardless of body region). Two additional comparisons were made: one comparing gene expression across samples of the anterior region sorted by the reproductive status of the animal (reproductive vs. non-reproductive, regardless of sex), and one comparing gene expression across samples of the midbody sorted by the reproductive status of the animal (reproductive vs. non-reproductive, regardless of sex).

All comparisons followed Trinity’s analysis pipeline (https://github.com/trinityrnaseq/trinityrnaseq/wiki/Post-Transcriptome-Assembly-Downstream-Analyses). Kallisto v0.45.0 [49] was used to estimate per condition transcript abundance, and differential expression analyses were performed with edgeR v3.40.2 [50, 51]. Transcripts were considered to be differentially expressed when FDR-corrected *p*-value < 0.001.

### Reproduction related transcripts

In order to identify differentially expressed transcripts related to reproduction, a custom-made R script (Supplementary File S2) was used to create an annotation file containing only those transcripts with differential expression. This new annotation file was then used to identify transcripts related to reproduction by using a keyword approach with a selection of keywords or sets of keywords chosen based on previous knowledge about syllid sexual maturation. For that, the annotation results of each unique transcript found to be differentially expressed in the appropriate comparisons were scanned for matches of terms associated with reproduction in the Gene Ontology results of assigned BLASTX hits as well as conserved protein domains. In addition to scanning the Gene Ontology results, we searched the identified conserved protein domains of each differentially expressed transcript for Pfam IDs related to protein families or molecules relevant for syllid sexual maturation. These include BMPs (PF00019), Fox proteins (PF00250), Hox proteins (PF00046, PF05920), methyl farnesoate (PF12248), Notch (PF01414, PF06816), relaxin (PF00049) and Wnt (PF00110). Occurrences of Gene ontology keywords and the mentioned Pfam IDs were then counted for each differential expression analysis.

## Results

After quality-based trimming of the sequenced reads, 631,223,163 read pairs were used for reference transcriptome assembly. The assembled transcriptome consisted of 710,144 transcripts clustered in 431,890 Trinity “genes” and was composed of 660,830,310 bases. The average transcript length was 930.56 bases and the N50 value was 1,932 bases. According to BUSCO’s completeness analysis, this reference transcriptome was 99.6% complete and had a duplication level of 94.8%. In order to perform differential expression analyses on the transcript level, a clustering process was implemented to reduce the number of redundant sequences and to improve the statistical analyses. Redundancy reduction resulted in a streamlined transcriptome with 516,683 transcripts and 411,292 Trinity “genes”, totaling 396,107,508 bases. The average transcript length after redundancy reduction was 766.64 bases and the N50 value was 1,330 bases. The clustered transcriptome has 99.5% BUSCO completeness and a duplication level of 76.9%. In addition, the BUSCO analysis of the unfiltered assembly supertranscripts was carried out to assess the true redundancy more accurately, which revealed 99.6% completeness and a 36.6% duplication level.

TransDecoder protein prediction from the clustered transcriptome resulted in 103,429 predicted protein sequences. Trinotate annotation using the assembled transcripts and the proteins predicted from them resulted in an Annotation Unit identification success of 20.63%. The assembled transcriptome, predicted proteins, and Trinotate annotation report can be found at https://github.com/gponz/Ramisyllis-kingghidorahi-transcriptome.

**Fig. 2:**
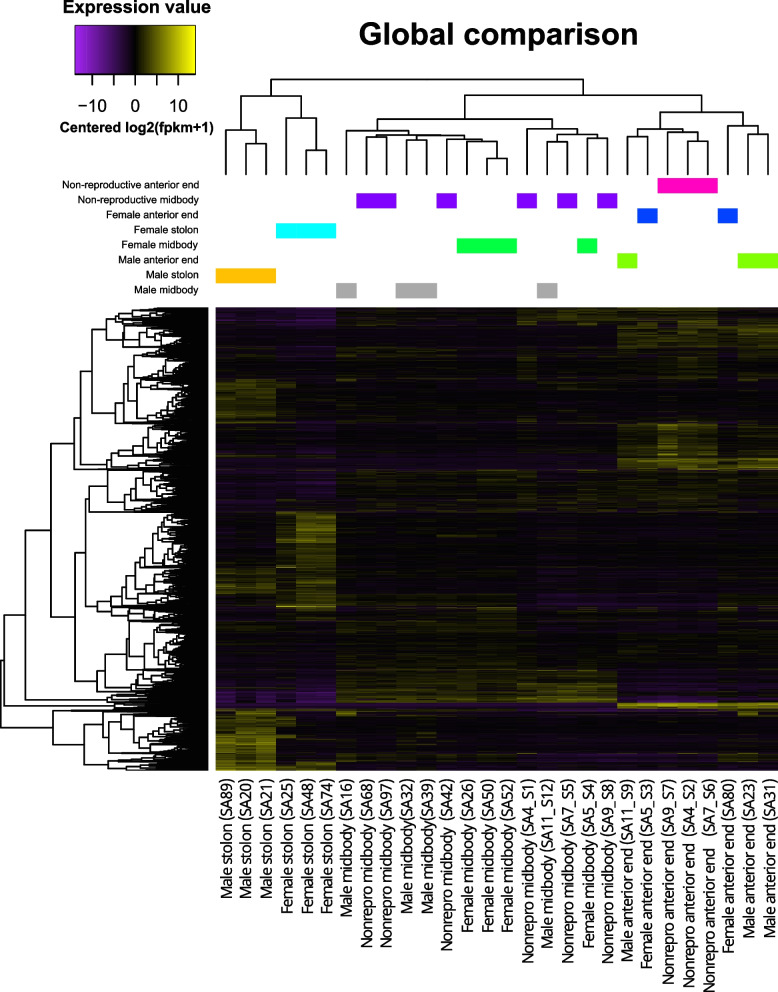
Global comparison of all samples characterized by their body region and sex conditions (columns; indicated by different colours) visualized by a heatmap representing the expression value [Centred log2(fpkm + 1)] of each differentially expressed transcript (lines) in each replicate. Yellow colours indicate higher expression values; purple colours indicate lower expression values. Trees on the top (samples) and left-hand side (transcripts) of each heatmap show hierarchical clustering based on similar expression patterns. Each column represents the expression pattern of a single replicate identified by an individual code (see Supplementary Files S4 and S5)

Six thousand seven hundred and six individual transcripts were found to have differential expression in at least one pairwise comparison (Supplementary File S3). The global differential expression analysis including all samples and considering the body region and sex they belong to showed 5,202 differentially expressed transcripts (Fig. 2; Supplementary Files S4 and S5). In this comparison, samples clustered by body region. The cluster of stolon samples was the most different in terms of gene expression of these 5,202 differentially expressed genes, while anterior end and midbody samples were more similar. Interestingly, within the stolon cluster, the samples are clearly grouped by sex. In contrast, within the anterior and midbody clusters, only the samples from anterior regions of non-reproducing animals were grouped together by sex.

**Fig. 3:**
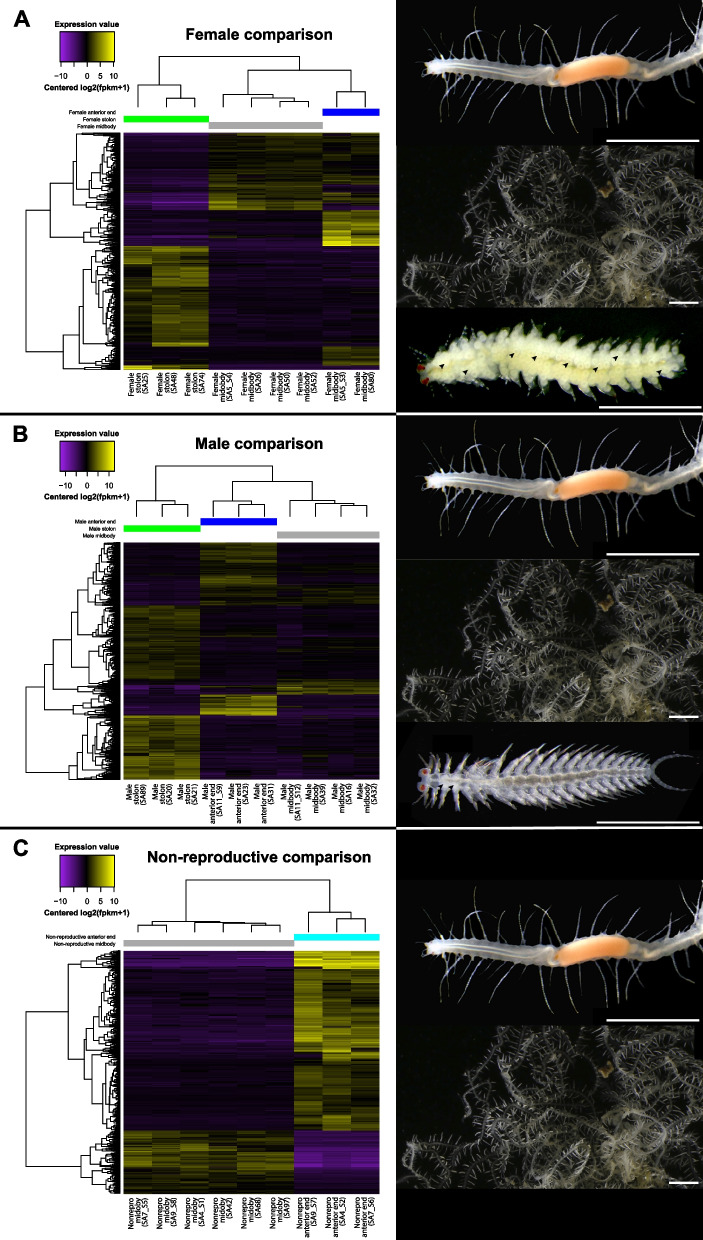
Within-sex comparison of female (**A**), male (**B**) and non-reproductive (**C**) specimens (columns; indicated by different colours) visualized by heatmaps representing the expression value [Centred log2(fpkm + 1)] of each differentially expressed transcript (lines) in each replicate. Yellow colours indicate higher expression values; purple colours indicate lower expression values. Trees on the top (samples) and left-hand side (transcripts) of each heatmap show hierarchical clustering based on similar expression patterns. Each column represents the expression pattern of a single replicate identified by an individual code (see Supplementary Files S6 to S11). Images on the right show the body regions included in each comparison: anterior end (top), midbody fragment (middle), and stolons (bottom). Images of the animals were modified from [37]. Arrowheads point towards individual oocytes (**A**) and sperm-filled segments (**B**). Scale bars: 2 mm (anterior end and midbody fragment), 500 µm (stolons)

In the differential expression analyses comparing the different body regions of each sex category, the female body region comparison revealed differential expression of 1,062 transcripts (Fig. 3A; Supplementary Files S6 and S7), the male body region comparison yielded 1,578 differentially expressed transcripts (Fig. 3B; Supplementary Files S8 and S9), and the non-reproductive body region comparison showed differential expression of 583 transcripts (Fig. 3C; Supplementary File S10 and 11). In all three comparisons, the samples cluster together by body region. Additionally, in both females and males, stolons are the body region with the most differentiated gene expression profile, showing little to no overlap in gene upregulation compared to the gene expression profile of the anterior end and midbody segments. Comparisons among body regions in the female and male analyses also revealed that the largest number of differentially expressed transcripts were upregulated in the stolon or the anterior end, while only a limited number of transcripts were upregulated in the midbody region. Similarly, in the non-reproductive animals, only a small proportion of the 583 differentially expressed transcripts were upregulated in the midbody, with the majority being upregulated in the anterior region (note that there are no stolons in these animals).

**Fig. 4:**
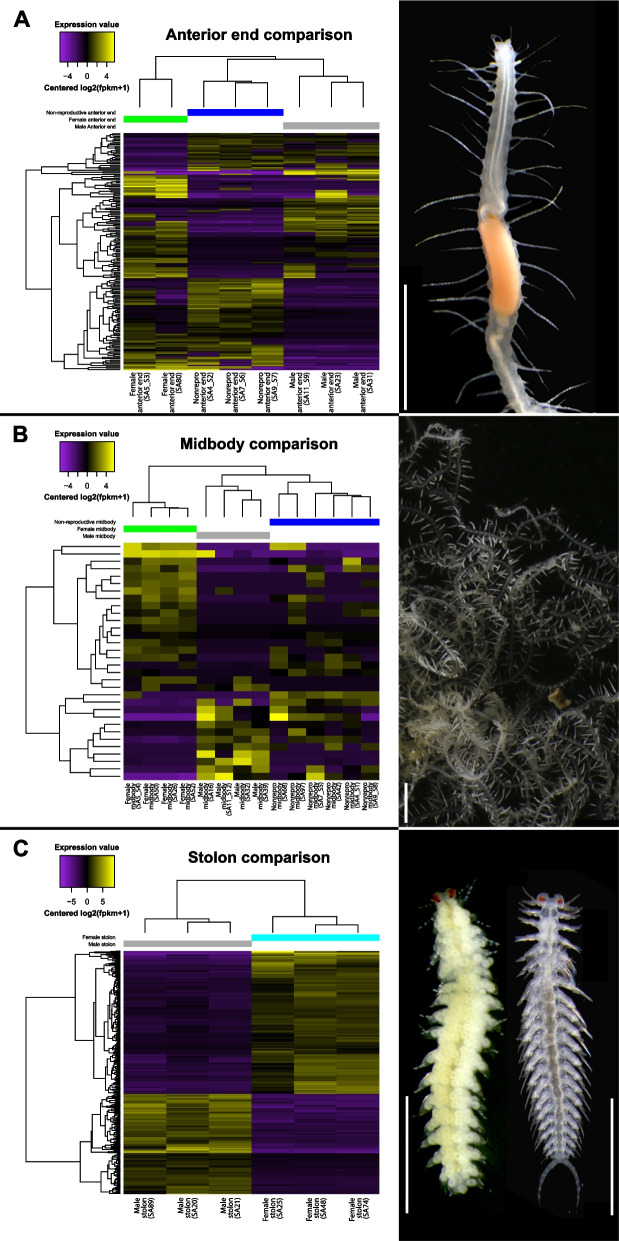
Across-sex comparison of the anterior end (**A**), the midbody region (**B**), and the stolons (**C**) (columns; indicated by different colours) visualized by heatmaps representing the expression value [Centred log2(fpkm + 1)] of each differentially expressed transcript (lines) in each replicate. Yellow colours indicate higher expression values; purple colours indicate lower expression values. Trees on the top (samples) and left-hand side (transcripts) of each heatmap show hierarchical clustering based on similar expression patterns. Each column represents the expression pattern of a single replicate identified by an individual code (see Supplementary Files S12, S13, S17, S18, S19 and S20). Images on the right show each body region: anterior end (**A**), midbody fragment (**B**), and female (left) and male (right) stolons (**C**). Images of the animals were modified from [37]. Scale bars: 2 mm (anterior end and midbody fragment), 500 µm (stolons)

As for the sex comparisons within each body region, in the anterior end, 175 transcripts were differentially expressed between male, female, and nonreproductive samples (Fig. 4A; Supplementary Files S12 and S13). The samples clustered together by sex condition with the female animals being the most different in their gene expression and the male and non-reproductive samples being more similar. This same comparison was also run without distinguishing male and female samples (i.e. comparing reproductive vs. non-reproductive animals), which resulted in 20 differentially expressed transcripts (Supplementary File S14; Supplementary Files S15 and S16), 13 of which overlapped with those shown in Fig. 4A. In the midbody region, 32 transcripts were differentially expressed across the three sex conditions with the samples clustering together by sex condition (Fig. 4B; Supplementary Files S17 and S18). In this analysis, the condition with the most distinctive gene expression pattern was the female midbody, with the male and the non-reproductive samples being more similar among them. Similar to the previous case, a second analysis without differentiating between males and females was run (reproductive vs. non-reproductive) with the midbody samples, though no differentially expressed transcripts were identified. Lastly, male and female stolons presented 456 differentially expressed transcripts, with the samples properly clustered together by sex and similar levels of up- and downregulation in both sex conditions (Fig. 4C; Supplementary Files S19 and S20).

**Fig. 5:**
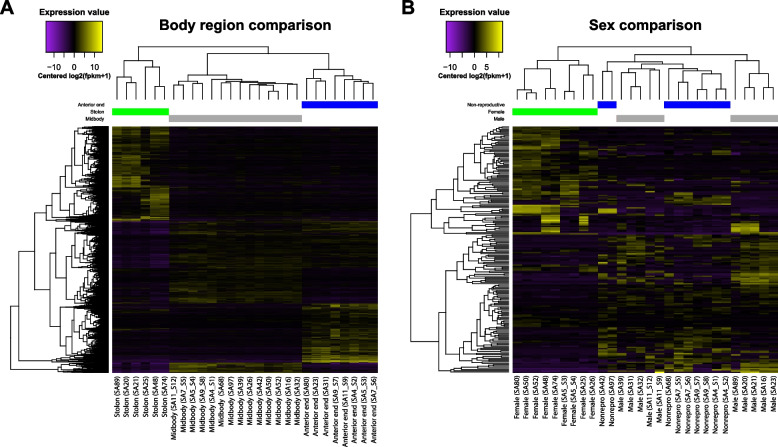
Comparison of differentially expressed transcripts across body regions without including sex categories (**A**) and across sex categories without including body region (**B**) visualized by heatmaps representing the expression value [Centred log2(fpkm + 1)] of each differentially expressed transcript (lines) in each replicate. Yellow colours indicate higher expression values; purple colours indicate lower expression values. Trees on the top (samples) and left-hand side (transcripts) of each heatmap show hierarchical clustering based on similar expression patterns. Each column represents the expression pattern of a single replicate identified by an individual code (see Supplementary Files S21 to 24)

The comparison of all samples considering only their body region condition (Fig. 5A; Supplementary Files S21 and S22) showed 4,120 differentially expressed transcripts, with samples clustering together by body region condition and the cluster of stolon samples being the most distinct. Of these 4,120 differentially expressed transcripts, 2,708 also appeared as differentially expressed in the global comparison shown in Fig. 2. Likewise, the comparison of all samples considering only their sex condition (Fig. 5B; Supplementary File S23 and S24) resulted in 198 differentially expressed transcripts. Female samples cluster together and are the most distinct in terms of gene expression pattern, while male and non-reproductive samples are intermingled. Of these 198 differentially expressed transcripts, 110 overlap with those of the global differential expression analysis. Considering the transcript overlap of both described analyses, the differential expressions of 2,800 out of the 5,202 differentially expressed transcripts in the global comparison likely stem from differences in either body region or sex, indicating that the remaining 2,402 differentially expressed transcripts (~ 46%) in the global analysis can be accounted for by differences in the different body regions between the sexes.

To assess potential bias in our results due to the pseudoreplication strategy described above, the sample correlation matrices resulting from each differential expression analysis were inspected to check whether samples coming from the same specimen had higher correlation values than completely independent replicates, especially in the case of sample pairs SA20-SA21 and SA50-SA52, which were from the same body region of the same specimen. In all cases, the correlation values of the pseudo-replicates were within the range of values presented by fully independent samples.

In order to quantify annotation results, pre-selected keywords were counted by searching BLASTx and Pfam Gene ontology output for keyword terms, as well as by scanning for the above-mentioned Pfam IDs in the annotation of the differentially expressed transcripts (Supplementary File S25). For each differential expression analysis, all unique transcripts with annotated keywords were then filtered (Supplementary File S26). This process allowed us to compare differences in the occurrence of specific processes or molecules between the pairwise differential expression analyses. The number of Gene Ontology keywords and Pfam ID matches in every unique annotated transcript of each differential expression pairwise comparison was counted and is presented in Table 1. Of the twenty-six sets of keywords (some related keywords were grouped together as they are considered here to be involved in the same processes), each keyword was associated with differentially expressed transcripts in at least one of the seven comparisons considered (anterior end, midbody, stolon, female, male, non-reproductive and global). The general trend was that broad categories tended to show a higher number of hits than narrower or very specific keywords, as was expected. For example, *Development*, *Embryonic*, *Morphogenesis*, *Cell cycle*, *Proliferation* and *Apoptosis* all had a comparatively high number of annotated, differentially expressed transcripts in all comparisons (*Notch* and *BMP*, both of which are also related to cell differentiation, were also well represented, albeit in a lesser degree). However, some other keywords or keyword sets are worth highlighting. The first such set is that of *Testis*/*Sperm*, which appeared in a fair amount of the annotated, differentially expressed transcripts in most comparisons (11.63% in anterior end, 11.7% in stolons, 8.41% in males, 8.81% in females, and 8.87% in non-reproductive specimens), while the keyword set *Oocyte*/*Oogeneis*/*Ovochymase* was less represented (0%, 2.13%, 1.94%, 3.52% and 1.61%, respectively). Interestingly, other keywords related to gonads or gametogenesis, like *Gametogenesis*, *Germline* and *Gonad*, had comparatively low numbers of hits. *Ecdysone*/*Molting* and *Fox* also showed very low numbers of hits. *Hox* showed the highest number of hits in comparisons of female, non-reproductive and stolon samples (5.32% in stolon, 3.96% in female, 4.84% in non-reproductive specimens). Another interesting group of keyword sets in relation to syllid sexual maturation and the development of sensory organs in the stolon are those related to hormonal activity (*Hormonal activity*/*Serotonin*/*Dopamine*), eye development (*Eye*/*Photo*/*Retinal*/*Retinol*/*Visual*/*Sox*/*Opsin*), and neurogenesis (*Neurogenesis*/*Neural tube*), all of which showed a clear pattern of higher percentages and numbers of hits in within-sex comparison. Lastly, *Sex* and *Circadian rhythm*, despite being often related to sexual maturation and reproduction in syllids, both had a comparatively small number of hits in all comparisons, with the only noteworthy exception being the 5.32% obtained in stolon samples.

**Table 1:** Annotation of pre-selected keywords in each of the depicted differential expression analyses

Keywords	Differential expression analysis													
	Anterior end		Midbody		Stolon		Male		Female		Non-reproductive		Global	
	N	%	N	%	N	%	N	%	N	%	N	%	N	%
Reproduction	0	0.00	0	0.00	0	0.00	5	1.62	1	0.44	1	0.81	6	0.56
Development	20	**46.51**	3	**50.00**	35	**37.23**	155	**50.16**	104	**45.81**	70	**56.45**	521	**48.24**
Sex	1	2.33	0	0.00	1	1.06	14	4.53	4	1.76	4	3.23	19	1.76
Gametogenesis	0	0.00	0	0.00	0	0.00	2	0.65	1	0.44	0	0.00	5	0.46
Oocyte/oogenesis/ovochymase	0	0.00	0	0.00	2	2.13	6	1.94	8	3.52	2	1.61	25	2.31
Testis/sperm	5	**11.63**	0	0.00	11	**11.7**	26	8.41	20	8.81	11	8.87	99	9.17
Germline	0	0.00	0	0.00	1	1.06	6	1.94	4	1.76	1	0.81	16	1.48
Relaxin	0	0.00	0	0.00	0	0.00	1	0.32	1	0.44	1	0.81	1	0.09
Hormonal activity/serotonin/dopamine	3	6.98	0	0.00	4	4.26	43	**13.92**	26	**11.45**	13	**10.48**	114	**10.56**
Morphogenesis	12	**27.91**	2	**33.33**	25	**26.60**	80	**25.89**	49	**21.59**	41	**33.06**	243	**22.50**
Gonad	0	0.00	0	0.00	4	4.26	7	2.27	5	2.2	1	0.81	21	1.94
Ecdysone/molting	0	0.00	0	0.00	1	1.06	4	1.29	1	0.44	1	0.81	7	0.65
Eye/photo/retinal/-ol/visual/sox/opsin	4	9.30	1	**16.67**	6	6.38	54	**17.48**	39	**17.18**	24	**19.35**	182	**16.85**
Neurogenesis/neural tube	1	2.33	0	0.00	5	5.32	31	**10.03**	18	7.93	15	**12.10**	69	6.39
Embryonic	4	9.30	1	**16.67**	11	**11.7**	33	**10.68**	19	8.37	15	**12.10**	109	**10.09**
Farnesoate	0	0.00	0	0.00	0	0.00	3	0.97	1	0.44	2	1.61	6	0.56
Proliferation	8	**18.60**	3	**50.00**	13	**13.83**	57	**18.45**	58	**25.55**	21	**16.94**	188	**17.41**
Circadian rhythm	0	0.00	0	0.00	5	5.32	8	2.59	9	3.96	1	0.81	35	3.24
Hox	0	0.00	0	0.00	5	5.32	3	0.97	9	3.96	6	4.84	29	2.69
Fox	0	0.00	0	0.00	0	0.00	3	0.97	1	0.44	3	2.42	10	0.93
WNT	5	**11.63**	0	0.00	8	8.51	13	4.21	10	4.41	7	5.65	50	4.63
Notch	1	2.33	0	0.00	1	1.06	25	8.09	21	9.25	13	**10.48**	47	4.35
BMP	5	**11.63**	1	**16.67**	2	2.13	9	2.91	3	1.32	6	4.48	25	2.31
Apoptosis	9	**20.93**	1	**16.67**	16	**17.02**	53	**17.15**	45	**19.82**	22	**17.74**	206	**19.07**
Cell cycle	4	9.30	2	**33.33**	19	**20.21**	21	6.8	33	**14.54**	6	4.84	126	**11.67**
Senescence	1	2.33	0	0.00	1	1.06	1	0.32	4	1.76	0	0.00	13	1.20
Number of annotated DE transcripts	43		6		94		309		227		124		1080	
Total number of DE transcripts	175		32		456		1578		1062		583		5202	

Count of keyword occurrences within the annotated, differentially expressed transcripts resulting from each differential expression analysis. Keywords are assigned by searching Gene Ontology annotation of BLASTx and Pfam output for the shown terms, as well as by scanning Pfam results for IDs of related protein domains. Percentages are given regarding the number of annotated, differentially expressed transcripts per analysis. Percentage values > 10% are highlighted. Note that the sum of occurrences of each keyword within the comparison does not add up to the number of annotated, differentially expressed values because not all annotated, differentially expressed transcripts are related to the keywords selected here and the same transcript can be related to more than one function or keyword. Percentages within each comparison do not add up to 100% for these reasons

## Discussion

The assembled transcriptome has a number of transcripts, average length, N50 values, and annotation ratio comparable to all other transcriptomes within the family [25, 27, 52–55]. Similarly, high completeness and high duplication within the transcriptome have also been repeatedly observed [25, 27, 52, 54]. Causes for artificial redundancy are alternative splicing, due to assemblers creating separate contigs for each protein isoform of the same gene, as well as contigs not representing isoforms stemming from variation in coverage, sequencing errors, or polymorphisms within a population [56]. Supertranscripts (gene-like sequences constructed by collapsing unique and common sequence regions among isoforms into a single linear sequence [57]) were created to minimize the influence of alternative splicing on redundancy. The remaining 36.6% duplication rate may still be at least partially artificial, possibly stemming from one or multiple of the aforementioned sources. True redundancy could also be a contributor to this duplication rate due to partial genome duplication in the species’ evolutionary history. Previous studies have proposed possible genome duplication events within Syllidae [58], supporting this as a hypothesis worth further investigation. To assess this possibility, future research should focus on chromosome number and genome architecture in syllids. Additionally, while clustering redundant sequences in the assembly reduced the duplication rate from 94.8% to 76.9%, a considerable proportion of the transcriptome remains redundant post-filtering, potentially impacting expression analysis.

In terms of differential expression, the most notable finding is that body region had a significantly stronger influence on the gene expression profile of each sample than sex. As shown in Fig. 2, when both factors were considered simultaneously, samples clustered primarily by body region, with sex contributing to a lesser and less distinct degree. This pattern aligns with expectations, as the anterior ends of all individuals, even during sexual maturation, likely exhibit greater similarity to each other than to other body regions due to their shared structure and function. When considering within-sex comparison, stolons are consistently identified as the body region with the most distinct expression pattern (Figs. 3A,B, and 5A). The stolons being the most different in their gene expression can probably be explained by the high concentration of region-specific biological functions they hold, including gametogenesis, but also the metamorphic changes they must undergo in order to be mature enough for independent swimming [6–10]. In females, both the stolons and the anterior end were characterized by considerable gene upregulation (Fig. 3A). The midbody, in turn, had fewer region-specific upregulated transcripts, which was also the case in the comparison of the anterior end and the midbody of non-reproductive individuals (Fig. 3C). In males, the overall pattern was similar to that of females, although the midbody segment has even fewer upregulated genes (Fig. 3B). Additionally, the relatively high number of differentially expressed transcripts in all within-sex comparisons further underscores the predominant influence of body region in determining gene expression, compared to the effect of sex.

In across-sex body region comparisons (Fig. 4), samples from the same body region clustered together based on sex in the differential expression analyses. This finding highlights the presence of sex-specific processes across all three body regions, even at an advanced stage of sexual development. In the context of *Ramisyllis*, this observation might have two interpretations. First, it may imply that the genetic mechanisms expressed in the anterior end that are involved in triggering the onset of sexual maturation and/or those involved in sex determination (which themselves could also be sex-specific) might keep working during the late stages of sexual development. Second, since it is known that sexually maturing *Ramisyllis* specimens often have stolons in different stages of their development [36, 37, 59], it could be that signaling from the anterior end is “continuous” and there are other clues or mechanisms involved in determining which posterior ends develop stolons at any given moment.

In relation to the anterior end (Fig. 4A), previous evidence in syllids [7, 11, 13, 15, 25] and other annelids [16–21, 60] have pointed toward an anteriorly-located sexual maturation control system in which female sex determination requires a more complex signaling process, which is consistent with the clustering of the sex conditions shown in Fig. 4A, where females are the most different. However, despite the differences in gene expression amongst the anterior ends, the expression patterns observed here in the anterior region of male and female *R. kingghidorahi* specimens do not show evidence of massively different sex-specific gene expression changes. This lack of largely noticeable differences in the extent of the gene expression changes in male and female anterior ends could be explained by at least three reasons. The first possible explanation might be that contrary to what has been proposed for other syllids and annelids, the mechanisms controlling sex determination in *R*. *kingghidorahi* could be similar in the extent of the gene expression changes they require in both sexes. The second possible explanation might be that the major determinants of sex determination could be non-genetic. For example, as discussed by Ponz-Segrelles et al. [27], it has been shown that some syllids are protogynous hermaphrodites, meaning they suffer a process of (often irreversible) masculinization as they age, with female-to-male sex changes being far more common than male-to-female transitions. The mechanisms underlying this masculinization process remain entirely unknown and, thus, while they likely involve changes in gene expression, the nature of these changes and how they would manifest in analyses such as those presented here remain uncertain. At the moment, it is not known whether *Ramisyllis* individuals go through sex changes throughout their lives [36, 37, 59] as many syllids do [13, 15, 61–67]. A third possible explanation is that methodological limitations may have prevented the detection of a potentially female-specific, more complex mechanism involving extensive differential gene expression in female anterior ends. For example, if the number of cells expressing key genes involved in this hypothetic mechanism were to be small, their presence in the total mix of RNA molecules of the body region sample (e.g. anterior end up to the end of the proventricle) could end up being too small for the methods used here to detect it. The true explanation for this apparent deviation from what has been reported in other syllids will need to be decided by future research.

Regarding stolons, Fig. 4C shows similar degrees of up- and downregulation for both sexes. These differences are likely to be partially due to differential changes in gene expression during gametogenesis e.g. [24, 53] and those related to external sexual dimorphism in stolons (the body of male stolons has a narrower body with larger parapodia and shows regionalization, while that of females is much thicker and evenly filled with oocytes) [37]. Morphogenetic changes could also explain why the across-sex stolon comparison has a much higher number of differentially expressed transcripts (456) than those of the anterior end (175) or the midbody (32). However, another possible (or complementary) explanation would be stolonial expression of sex-specific regulatory genes involved in the control of reproduction as proposed by [25]. In this latter sense, our results showing differential expression between male and female stolons are consistent with the existence of signaling molecules produced in the stolon and involved in regulating sexual maturation, probably through a negative feedback loop modulating the production of methylfarnesoate and/or other anteriorly-produced hormones [25].

For midbody fragments (Fig. 4B), female samples exhibited the most distinct expression pattern, while males and non-reproducing individuals were more similar to each other. Currently, no clear biological explanation exists for this result, suggesting that methodological factors, such as the challenge of selecting midbody samples while minimizing the inclusion of posterior ends, may have influenced the findings. Similarly, female samples exhibited the most distinct gene expression overall when compared to male and non-reproductive samples (Fig. 5B). These findings may suggest that key gene regulatory processes are female-specific, or they could be attributed to methodological factors, such as the inability to determine the sex of the animals before signs of stolon development, despite the potential onset of sex determination and gonad development [68].

The expression of genes associated with functions known or expected to be related to sexual maturation was significantly affected by the relatively low success in the annotation process. This limitation is likely due to the under-representation of most annelids in available databases, which are predominantly populated with data from more commonly studied model organisms. This problem is also likely to be partly responsible for the differentially expressed genes being more often linked to broad functional categories like *Development* or *Morphogenesis*, rather than to more specific ones. If the physiology of reproduction in annelids is poorly known, fewer specific genes can be expected. Nevertheless, the results presented in Table 1 indicate that, although hormonal changes occur in the body of *R. kingghiidorahi* specimens during sexual maturation, the differences across sex conditions for each body region are limited. This suggests that few sex-specific changes in hormonal activity were detected. Moreover, despite it being central to the current model of the molecular control of sexual maturation in syllids, our results showed very few differential expression of genes related to *Farnesoate*. This lack of *Farnesoate*-related results could point to a difference in the mechanism regulating sexual maturation in this species and the one proposed by Álvarez-Campos et al. [25]. However, given that methylfarnesoate has been proposed to be an early signaling molecule in the maturation process [25], it is perhaps more likely that this difference, as well as the overall lack of sex-specific changes in hormonal activity, is due to the animals being in different stages of the stolonization cycle than those previously studied. Interestingly, a similar explanation could be behind the surprising lack of results in the categories more directly related to gonad and gamete development like *Gametogenesis*, *Oocyte/oogenesis/ovochymase*, *Testis/sperm*, *Germline*, or *Gonad*. Previous studies have noted that gene expression patterns related to gonad and gamete development change during sexual maturation in various annelids [69–71]. Since the animals used in this study were primarily in later stages of sexual maturation, with gametes already formed, it is possible that more time-sensitive studies are needed to fully elucidate the mechanisms involved.

Other noteworthy results include changes in the expression of genes related to the eyes, which was responsible for a significant percentage of the gene expression changes in the global and across-sex comparisons. These results indicate that, while eye development occurs locally in the anterior end and stolons, there is only little variation in eye-related gene expression between the different sexes. Although notably understudied in stolons when compared to their regular adult counterpart, eyes and brains are a prominent feature of stolons and yet, there is no information about the molecular basis of their development or functioning. Similarly, studies on nervous system development in basally branching annelids have revealed ancestral and convergent features of the brain and ventral nerve cord of major annelid groups [72–75]. However, whether these commonalities are also at play in stolonial brains has not been assessed. The results provided here are the first step towards a better understanding of the similarities between stolonial and regular eyes and brains at the developmental and molecular levels.

Finally, in regard to genes potentially linked to the metamorphic changes associated with stolonization, which might include *Wnt* or *Hox* genes [76–88], but also those under *Ecdysone/molting* [89, 90] based on what is known about segmentation and axis polarity establishment in adult annelids, our results showed only little evidence of these mechanisms being active at the studied developmental stages. This result is in agreement with what has been recently found by Nakamura et al. [24], which showed no evidence of differential expression of Hox genes in stolon-bearing and stolon-free individuals or different stages of stolon development in *Megasyllis nipponica*, which led the authors to conclude that, despite showing typically-anterior structures, stolons do not have a specific segment identity fingerprint.

As outlined, the results presented in this article demonstrate that stolonization in *R. kingghidorahi* is associated with body-region-specific and sex-specific gene expression changes. These include genes related to cell cycle regulation, as well as developmental and signaling processes involved in stolonization, such as eye development, hormonal activity, and sperm development, affecting both the stolons and the anterior end. Notably, this is consistent with all previously published results suggesting that reproduction in schizogamous syllids is under still-poorly-understood hormonal controlling mechanisms involving the anterior end. However, the branched body of *Ramisyllis* specimens still raises further questions about how these animals regulate their reproduction. For example, while scissiparous syllids only produce one stolon at their single posterior end, *Ramisyllis* specimens must induce stolonization at hundreds of posterior ends with their single anterior end. Yet, the anterior end of *Ramisyllis* specimens is anatomically similar to those of other syllids [38]. How can such a tiny brain regulate stolonization at so many posterior ends thousands of segments away from the prostomium? And even then, why do only some posterior ends produce stolons at any given time? If the stolonization-inducing mechanisms are acting, why do not all posterior ends stolonize? Is this process related or affecting the sponges in any way? And why is it possible to simultaneously find stolons in all maturation stages within the same stolonizing specimen while other specimens show no sign of stolonization even when collected at the same location and time (i.e. same environmental signals)? The results presented here clearly show that stolonization in *R. kingghidorahi* involves gene expression changes comparable to those of scissiparous syllids, including signaling from the anterior end. Yet, despite the findings of this study and those by Álvarez-Campos et al., Ponz-Segrelles et al. and Nakamura et al. [24, 25, 27], there is still much to be learned regarding the differential control of sexual maturation in male and female syllids and more physiological research is needed to identify the controlling mechanisms of stolonization-associated metamorphic changes and the signaling pathways involved in sexual maturation and sex determination in both linear and branched species.

## Conclusion

We provide the first assembled transcriptome of any branched syllid and any member of the Ribbon Clade of Syllinae. Analysis of the assembly supertranscripts reveals a significant degree of redundancy, suggesting the possibility of partial genome duplication in *Ramisyllis*. Comparative transcriptomics indicates that gene expression patterns in this species are generally consistent with the mechanisms regulating sexual maturation in unbranched schizogamic syllids. Differential expression analyses reveal greater differences in expression levels between body regions than between sexes, although there is clear evidence for sex-specific processes being active in all body regions. All analyses consistently indicate that stolons have the most different gene expression profile among body regions, likely due to highly region-specific processes regarding gametogenesis and metamorphosis. Likewise, females are found to have the most different gene expression profile among sexes, hinting at the presence of sex-specific molecular mechanisms. Within-sex comparisons reveal distinct gene expression patterns in both the stolons and anterior end, while across-sex comparisons show significant differences in the stolons, but less pronounced differences in the anterior end. These findings underscore the crucial roles of the anterior end and stolons in gene regulation, yet challenge prior expectations that predicted substantial sex-specific differences in the gene expression profiles of the anterior end due to a sex-specific, anteriorly-located control system for sexual maturation.

Annotation of Pfam and Gene Ontology terms showed low levels of detection for conserved protein families or signaling pathways, though within-sex analyses revealed a substantial level of upregulation for transcripts related to eye development, an understudied component of stolon development. Most key aspects of the reproductive endocrinology of branched syllids, and syllids in general, are still poorly known and increased efforts in this sense are needed to reveal the full complexity of syllid reproductive biology.

## Supplementary Information

Supplementary Material 1.

Supplementary Material 2.

Supplementary Material 3.

Supplementary Material 4.

Supplementary Material 5.

Supplementary Material 6.

Supplementary Material 7.

Supplementary Material 8.

Supplementary Material 9.

Supplementary Material 10.

Supplementary Material 11.

Supplementary Material 12.

Supplementary Material 13.

Supplementary Material 14.

Supplementary Material 15.

Supplementary Material 16.

Supplementary Material 17.

Supplementary Material 18.

Supplementary Material 19.

Supplementary Material 20.

Supplementary Material 21.

Supplementary Material 22.

Supplementary Material 23.

Supplementary Material 24.

Supplementary Material 25.

Supplementary Material 26.

## Data Availability

Raw RNA reads used in this study are available at the NCBI Sequence Read Archive (SRA) under BioProject ID PRJNA772756 with individual BioSample accession codes: SAMN22415819; SAMN22415820; SAMN22415821; SAMN22415822; SAMN22415823; SAMN22415824; SAMN22415825; SAMN22415826; SAMN22415827; SAMN22415828; SAMN22415829; SAMN22415830; SAMN22415831; SAMN22415832; SAMN22415833; SAMN22415834; SAMN22415835; SAMN22415836; SAMN43389008; SAMN43389087; SAMN43389149; SAMN43389175; SAMN43389409; SAMN43390836; SAMN43392463; SAMN43392468; SAMN43392745; and SAMN43393325.

## Abbreviations

BMP: Bone morphogenetic protein
Fox: Forkhead box proteins
Hox: Homeobox proteins
SIH: Stolonization-Inhibiting Hormone
SOX: SRY-related HMG-box genes
SPH: Stolonization-Promoting Hormone
