# Supplementary material for: Sex-specific differential gene expression during stolonization in the branching syllid Ramisyllis kingghidorahi (Annelida, Syllidae)
Source: BMC Genomics. 2025 Apr 25;26:405. doi: 10.1186/s12864-025-11587-w (PMC12023644; doi:10.1186/s12864-025-11587-w)
Supplement: Supplementary file 1 — Supplementary Material 1. [file 12864_2025_11587_MOESM1_ESM.docx]

**Supplementary File 1.** Samples sequenced for comparative transcriptomics.

| Species | Sample voucher | Specimen | Sex | Sample type |
| --- | --- | --- | --- | --- |
| *Ramisyllis* *kingghidorahi* | SA16 | Specimen 1 | Male | Midbody fragments |
| *Ramisyllis* *kingghidorahi* | SA32 | Specimen 2 | Male | Midbody fragments |
| *Ramisyllis* *kingghidorahi* | SA39 | Specimen 3 | Male | Midbody fragments |
| *Ramisyllis* *kingghidorahi* | SA11_S12 | Specimen 4 | Male | Midbody fragments |
| *Ramisyllis* *kingghidorahi* | SA20 | Specimen 1 | Male | Stolons |
| *Ramisyllis* *kingghidorahi* | SA21 | Specimen 1 | Male | Stolons |
| *Ramisyllis* *kingghidorahi* | SA89 | Specimen 5 | Male | Stolons |
| *Ramisyllis* *kingghidorahi* | SA23 | Specimen 1 | Male | Anterior region |
| *Ramisyllis* *kingghidorahi* | SA31 | Specimen 2 | Male | Anterior region |
| *Ramisyllis* *kingghidorahi* | SA11_S9 | Specimen 4 | Male | Anterior region |
| *Ramisyllis* *kingghidorahi* | SA26 | Specimen 6 | Female | Midbody fragments |
| *Ramisyllis* *kingghidorahi* | SA50 | Specimen 7 | Female | Midbody fragments |
| *Ramisyllis* *kingghidorahi* | SA52 | Specimen 7 | Female | Midbody fragments |
| *Ramisyllis* *kingghidorahi* | SA5_S4 | Specimen 8 | Female | Midbody fragments |
| *Ramisyllis* *kingghidorahi* | SA25 | Specimen 6 | Female | Stolons |
| *Ramisyllis* *kingghidorahi* | SA48 | Specimen 7 | Female | Stolons |
| *Ramisyllis* *kingghidorahi* | SA74 | Specimen 9 | Female | Stolons |
| *Ramisyllis* *kingghidorahi* | SA80 | Specimen 9 | Female | Anterior region |
| *Ramisyllis* *kingghidorahi* | SA5_S3 | Specimen 8 | Female | Anterior region |
| *Ramisyllis* *kingghidorahi* | SA42 | Specimen 10 | Non-reproductive | Midbody fragments |
| *Ramisyllis* *kingghidorahi* | SA68 | Specimen 11 | Non-reproductive | Midbody fragments |
| *Ramisyllis* *kingghidorahi* | SA97 | Specimen 12 | Non-reproductive | Midbody fragments |
| *Ramisyllis* *kingghidorahi* | SA4_S1 | Specimen 13 | Non-reproductive | Midbody fragments |
| *Ramisyllis* *kingghidorahi* | SA7_S5 | Specimen 14 | Non-reproductive | Midbody fragments |
| *Ramisyllis* *kingghidorahi* | SA9_S8 | Specimen 15 | Non-reproductive | Midbody fragments |
| *Ramisyllis* *kingghidorahi* | SA4_S2 | Specimen 13 | Non-reproductive | Anterior region |
| *Ramisyllis* *kingghidorahi* | SA7_S6 | Specimen 14 | Non-reproductive | Anterior region |
| *Ramisyllis* *kingghidorahi* | SA9_S7 | Specimen 15 | Non-reproductive | Anterior region |
