# Supplementary material for: Sex-specific differential gene expression during stolonization in the branching syllid Ramisyllis kingghidorahi (Annelida, Syllidae)
Source: BMC Genomics. 2025 Apr 25;26:405. doi: 10.1186/s12864-025-11587-w (PMC12023644; doi:10.1186/s12864-025-11587-w)
Supplement: Supplementary file 14 — Supplementary Material 14. [file 12864_2025_11587_MOESM14_ESM.pdf]

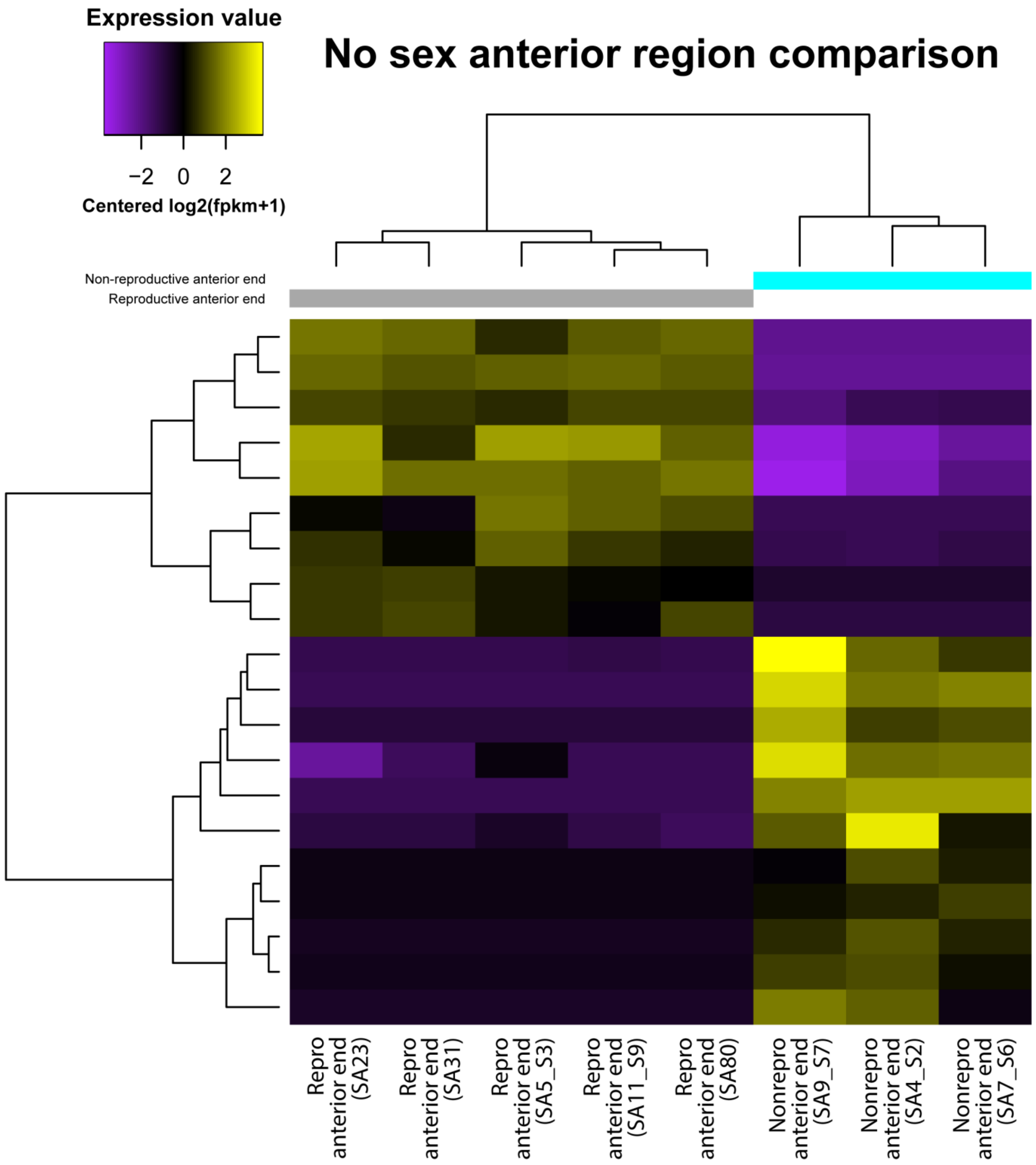

Supplementary File S14. Comparison of anterior-end samples grouped by sex condition (columns; indicated by different colours) visualized by a heatmap representing the expression value [Centred log2(fpkm+1)] of each differentially-expressed transcript (lines) in each replicate. Yellow colours indicate higher expression values; purple colours indicate lower expression values. Trees on the top (samples) and left-hand side (transcripts) of each heatmap show hierarchical clustering based on similar expression patterns. Each column represents the expression pattern of a single replicate identified by an individual code (see Supplementary Files S15 and S16).
